# Rapidly Progressive Cardiac Tamponade in Uncontrolled Rheumatoid Arthritis Complicated by Diabetic Ketoacidosis

**DOI:** 10.7759/cureus.96976

**Published:** 2025-11-16

**Authors:** Turna Zaman, Rafid Mustafa, Sultana Jannatun Nahar, G K M Rashik Uzzaman, Cornelius Fernandez James

**Affiliations:** 1 Acute Medicine, United Lincolnshire Hospitals NHS Trust, Boston, GBR; 2 Internal Medicine, Hull University Teaching Hospitals NHS Trust, Boston, GBR; 3 General Medicine, United Lincolnshire Hospitals NHS Trust, Boston, GBR; 4 General Medicine, United Lincolnshire Hospitals NHS Trust, Lincolnshire, GBR; 5 Endocrinology and Metabolism, Pilgrim Hospital, Boston, GBR

**Keywords:** cardiac arrest, cardiac tamponade, diabetic ketoacidosis, pericardial drain, pericardial effusion, rheumatoid arthritis

## Abstract

Rheumatoid arthritis (RA) can involve extra-articular organs, including the heart, with pericardial effusions being common but rarely progressing to cardiac tamponade. We report a 42-year-old female with uncontrolled RA, markedly elevated anti-cyclic citrullinated peptide (anti-CCP) and rheumatoid factor (RF), and type 2 diabetes mellitus, who presented with severe chest pain, shortness of breath, and diabetic ketoacidosis. Rapid accumulation of a large pericardial effusion led to cardiac tamponade, requiring emergency pericardial drainage during a peri-arrest state. Despite aggressive critical care, she developed hypoxic brain injury and multi-organ dysfunction, ultimately leading to death. This case highlights the importance of early recognition of cardiac involvement in RA, the potential severity of extra-articular manifestations even in the absence of active joint disease, and the need for prompt intervention in life-threatening complications.

## Introduction

Rheumatoid arthritis (RA) is a chronic autoimmune inflammatory disorder that primarily affects synovial joints but can also involve multiple extra-articular organs, including the cardiovascular system [1,2]. Cardiac involvement in RA is often clinically silent, yet it contributes significantly to morbidity and mortality, with pericardial effusion being a common manifestation, whereas cardiac tamponade is an uncommon manifestation [3]. Most cases of pericardial effusion in RA are asymptomatic and detected incidentally. However, in rare instances, pericardial effusion can present as the initial manifestation, even preceding articular symptoms [1,2]. We report a case of rapidly progressive pericardial effusion leading to cardiac tamponade in a patient with uncontrolled RA, highlighting the diagnostic and therapeutic challenges of this rare but potentially life-threatening presentation.

## Case presentation

A 42-year-old female presented to the accident and emergency (A&E) department with a three-day history of left-sided chest pain. The pain was described as pressure-like, radiating to the left arm and back, with a severity of 10/10, and was associated with exertional shortness of breath. She also reported two episodes of non-bilious, non-bloody vomiting containing clear fluid, as well as a recent history of cough and coryzal symptoms, but denied fever or urinary complaints. Notably, she had attended A&E the previous day, where she was referred to the same day emergency care (SDEC) unit and discharged with a working diagnosis of musculoskeletal chest pain and viral illness, and prescribed naproxen and omeprazole. Her past medical history included RA with markedly elevated anti-cyclic citrullinated peptide (anti-CCP) antibodies (>500) and rheumatoid factor (RF) (69 IU/mL), type 2 diabetes mellitus (T2DM), and essential hypertension. Her regular medications consisted of methotrexate 20 mg orally once weekly with folic acid supplementation for RA, in addition to oral hypoglycemic agents for T2DM. She has been taking methotrexate for six months prior to the presentation.

**Figure 1:**
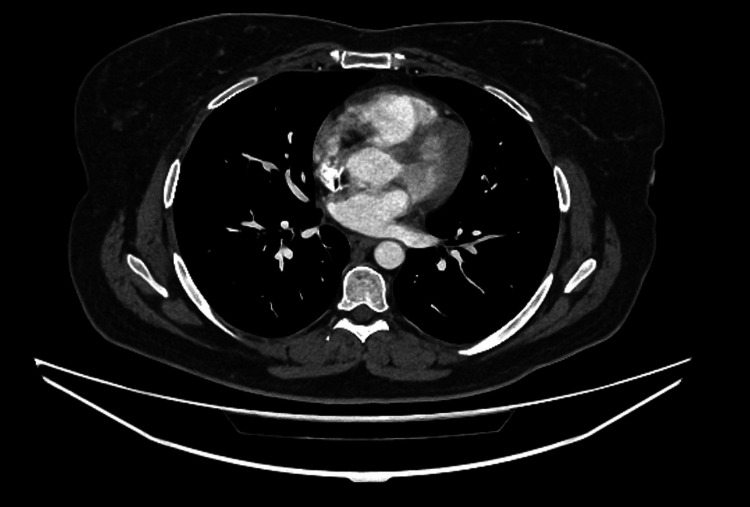
Computed tomography pulmonary angiography (CTPA) sagittal view.

**Figure 2:**
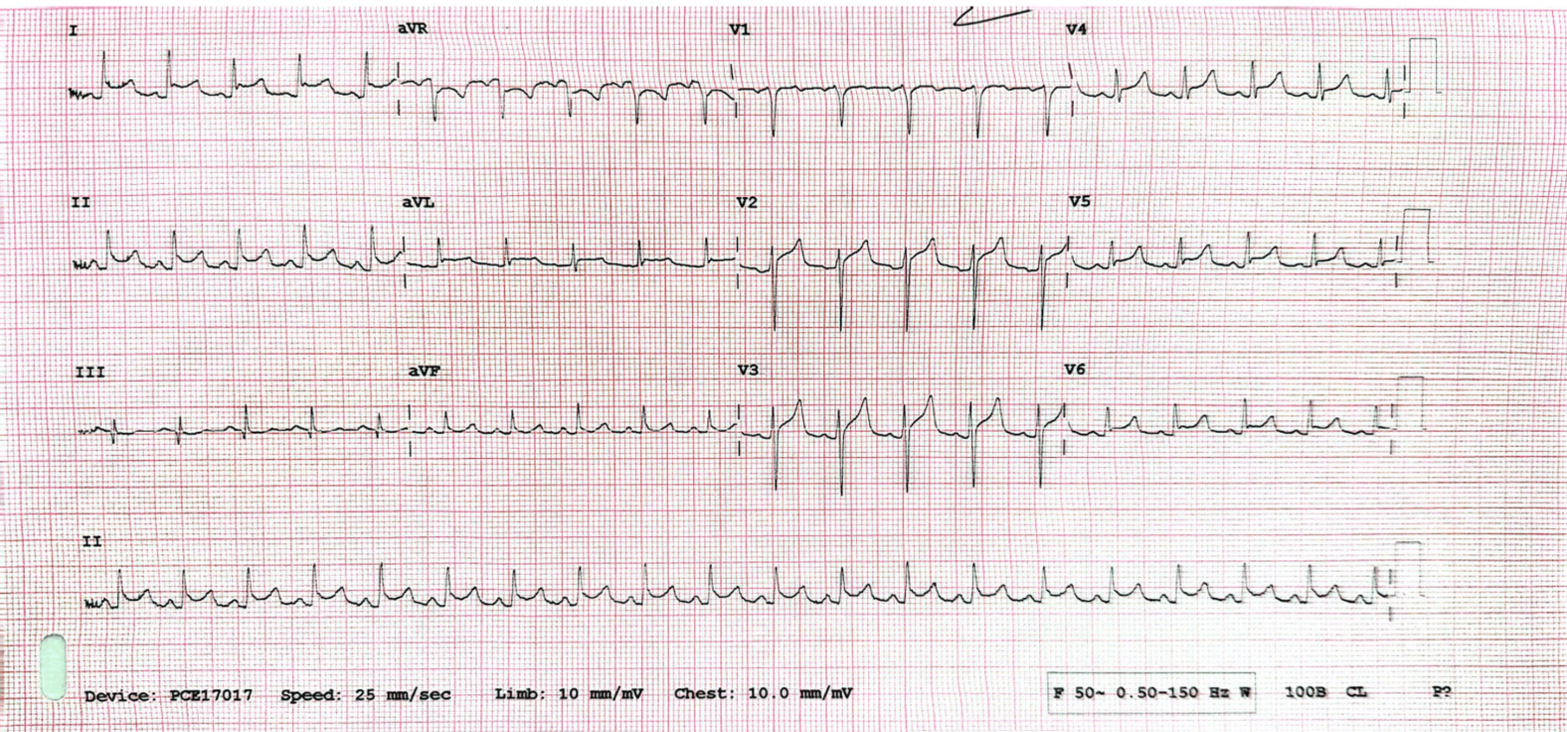
Electrocardiography (ECG). Electrocardiography revealed global saddle-shaped ST-segment elevation with PR-segment depression.

During admission, systemic examination findings were unremarkable. However, the patient subsequently deteriorated clinically with a National Early Warning Score (NEWS) of 7. Her vital signs at that time were as follows: heart rate of 113 beats per minute, respiratory rate of 30 breaths per minute, temperature of 36.4°C, and oxygen saturation maintained at 94% with 15 L of supplemental oxygen. Blood investigations revealed a markedly elevated C-reactive protein (CRP) of 245 mg/L, white cell count (WCC) of 21.1 × 10⁹/L (neutrophils 19.3 × 10⁹/L), estimated glomerular filtration rate (eGFR) of 45 mL/min/1.73 m², troponin T at 45 ng/L, and D-dimer at 1029 ng/mL (Table 1). She was clinically and biochemically euthyroid. There was no evidence of pulmonary embolism on the computed tomography pulmonary angiography (CTPA) (Figure 1), and chest radiography was unremarkable. Electrocardiography (ECG) revealed global saddle-shaped ST-segment elevation with PR-segment depression (Figure 2). Venous blood gas analysis showed a pH of 7.0 and bicarbonate (HCO₃⁻) of 10.4 mmol/L. Capillary blood glucose was 25 mmol/L, and blood ketones were 5.4 mmol/L. Based on the ECG findings, she was treated for pericarditis, and given her metabolic profile, a diagnosis of diabetic ketoacidosis (DKA) was also established.

**Table 1:** Blood investigation results.

Test	Result	Unit	Normal reference range
C-reactive protein (CRP)	245	mg/L	<5 mg/L
White cell count (WCC)	21.1	×10⁹/L	4.0 – 11.0 ×10⁹/L
Neutrophils	19.3	×10⁹/L	2.0 – 7.5 ×10⁹/L
Estimated glomerular filtration rate (eGFR)	45	mL/min/1.73 m²	≥90 (normal), 60–89 (mild ↓), 30–59 (moderate ↓)
Troponin	45	ng/L	<14 ng/L (assay dependent)
D-dimer	1029	ng/mL	<500 ng/mL

The cardiology team initially reviewed the patient and recommended continuous cardiac monitoring, adequate analgesia, and a planned echocardiogram. Following the diagnosis of DKA, the endocrine team initiated the DKA protocol alongside intravenous antibiotics. As the patient continued to be breathless with tachycardia and tachypnoea despite improvement of DKA, an alternative aetiology for her symptoms was sought. She was transferred to the intensive therapy unit (ITU) for close monitoring. Given the worsening clinical status, cardiology was reconsulted and advised an urgent bedside echocardiogram in the ITU.

**Figure 3:**
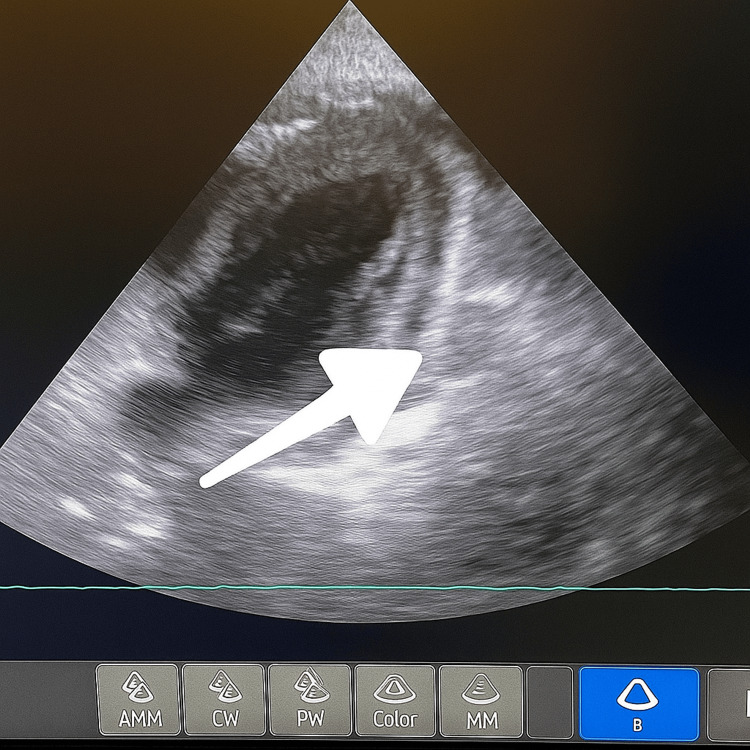
Bedside echocardiogram. The arrow indicates pericardial effusion.

A bedside echocardiogram demonstrated a 2.1 cm circumferential pericardial effusion. There was evidence of right ventricular diastolic flattening, and the left ventricular function appeared impaired, although it was uncertain whether this represented a true systolic dysfunction or an impaired expansion secondary to tamponade physiology. Subcostal views were unobtainable; however, adequate parasternal and apical views were achieved (Video 1 and Figure 3). Notably, the CTPA performed the previous day had shown no significant pericardial effusion, indicating rapid accumulation. Taken together, the clinical and imaging findings were consistent with cardiac tamponade, and the patient was planned for urgent pericardial drainage via an apical approach.

**Video 1:** Bedside echocardiogram. A bedside echocardiogram demonstrated a 2.1 cm circumferential pericardial effusion. There was evidence of right ventricular diastolic flattening, and the left ventricle appeared impaired, although it was uncertain whether this represented true systolic dysfunction or impaired expansion secondary to tamponade physiology. Subcostal views were unobtainable; however, adequate parasternal and apical views were achieved.

The patient developed cardiogenic shock and required noradrenaline infusion under central venous pressure (CVP) monitoring. Given the patient’s peri-arrest state, it was determined that the benefits of draining the pericardial effusion outweighed the procedural risks. As the patient became increasingly agitated, intubation was planned to facilitate the intervention; however, during preparations, she suffered a cardiac arrest with a non-shockable rhythm, with a low-flow state lasting 14 minutes. During the arrest, the cardiology consultant inserted a pericardial drain, with correct placement confirmed on echocardiography. Return of spontaneous circulation (ROSC) was achieved following the procedure. Following the arrest, she was kept intubated and ventilated, sedated with propofol and remifentanil, continued on the DKA protocol, maintained on noradrenaline targeting a mean arterial pressure (MAP) of 65 mmHg, and required continuous venovenous hemodialysis (CVVHD).

**Figure 4:**
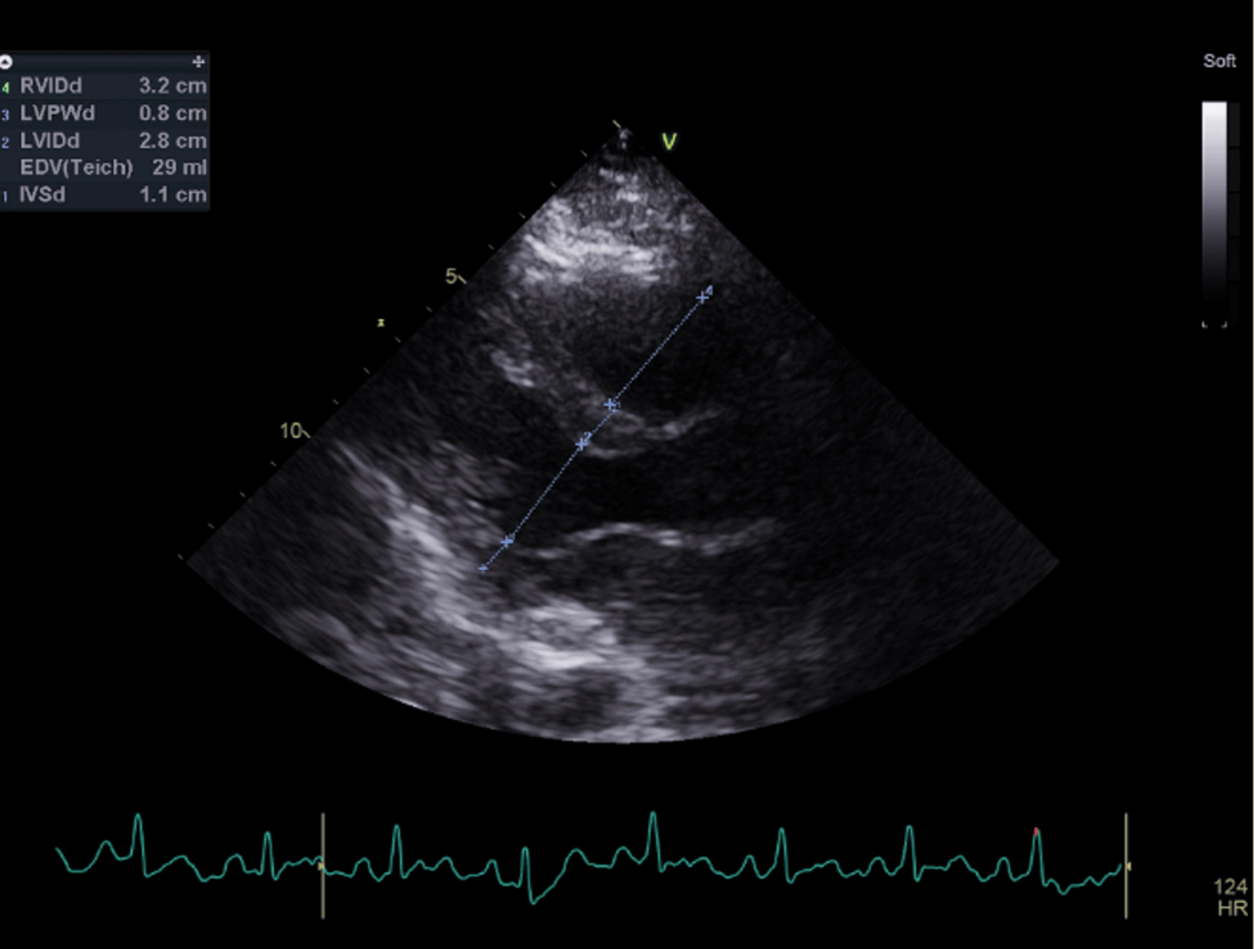
Echocardiography post pericardial drain. Echocardiogram demonstrated no significant pericardial effusion on parasternal and apical views, while subcostal views were difficult to obtain. A small amount of residual fluid was noted (<0.4 cm).

Post-arrest blood tests revealed deranged liver function, and gastroenterology advised this was likely secondary to ischemic hepatitis and expected to be self-resolving. A focused bedside echocardiogram demonstrated no significant pericardial effusion on parasternal and apical views, while subcostal views were difficult to obtain. A small amount of residual fluid was noted (<0.4 cm) (Figure 4), with no evidence of hemodynamic compromise, and removal of the pericardial drain was planned. Examination revealed dark discoloration of the hands and feet, likely due to poor perfusion secondary to inotropes. She was subsequently weaned off inotropes and sedation but remained critically unwell.

The rheumatologist reviewed the patient in the ITU and suggested that the pericardial effusion was likely related to underlying RA. They also noted the possibility of non-compliance with methotrexate, raising the likelihood that the pericardial effusion and cardiac tamponade represented a complication of uncontrolled RA. Review of clinic letters indicated that the patient had experienced recurrent flu-like symptoms and had been intermittently off methotrexate. She was stabilized from DKA and transitioned to a variable rate intravenous insulin infusion (VRIII) in combination with long-acting insulin.

Analysis of the pericardial fluid demonstrated moderate white blood cells, no organisms, and negative cultures. Histology revealed a cellular specimen containing numerous neutrophils and macrophages, with inconspicuous or absent mesothelial cells and no malignant cells. The overall impression was pericardial effusion secondary to acute inflammation.

**Figure 5:**
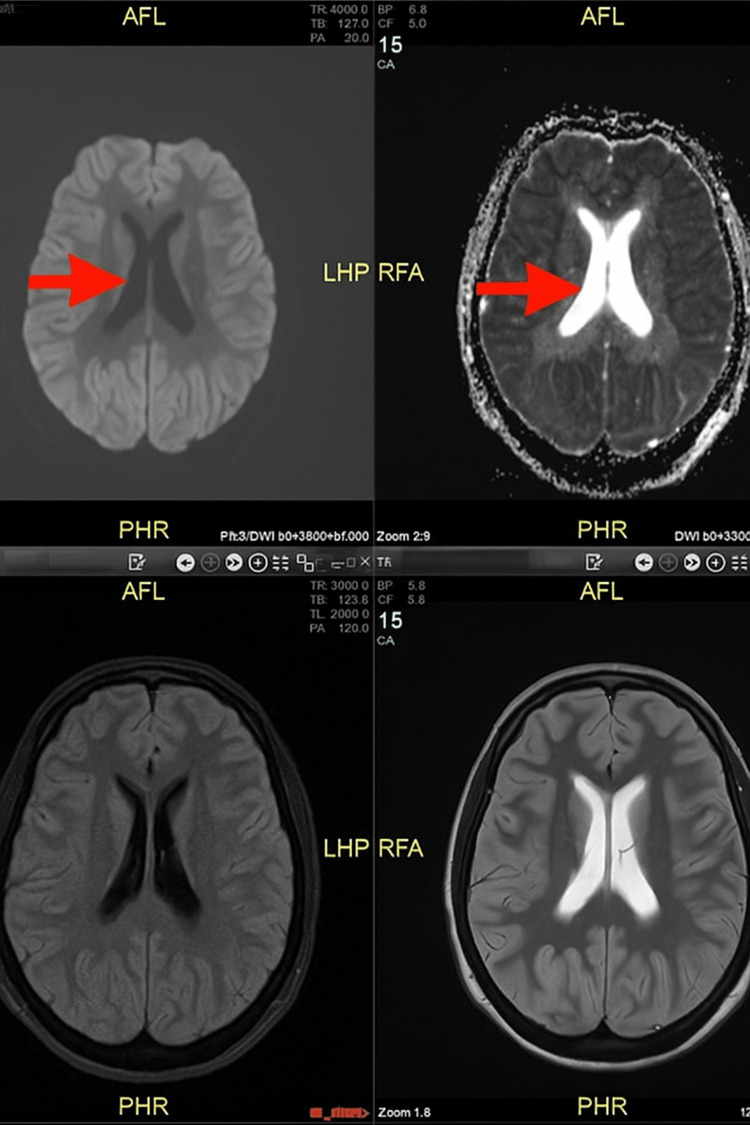
Magnetic resonance imaging (MRI) of the brain. MRI of the brain demonstrated widespread ischemic changes involving the cerebral cortex, with subtle but definite restricted diffusion, likely secondary to hypoxia.

Despite being off sedation, the patient remained unresponsive. Magnetic resonance imaging (MRI) of the brain demonstrated widespread ischemic changes involving the cerebral cortex, with subtle but definite restricted diffusion, likely secondary to hypoxia (Figure 5). Despite maximal critical care support, her condition continued to deteriorate. A best-interest decision was made, and a do-not-attempt cardiopulmonary resuscitation (DNACPR) decision and ceiling of therapy were established. She was subsequently transitioned to palliative care, focused on comfort. She later developed asystole and succumbed to it.

## Discussion

RA is a chronic autoimmune condition that primarily affects individuals in their third and fourth decades of life. Its annual incidence is estimated between 0.3% and 1.0%, with an overall prevalence of approximately 1% [1]. The disease is characterized by symmetrical synovial joint inflammation, which, if inadequately treated, can lead to cartilage destruction, bone erosion, deformity, and irreversible disability [1]. In addition to joint involvement, systemic features such as fever, weight loss, and fatigue are common, reflecting the systemic nature of RA. Extra-articular manifestations are well recognized and may involve the skin, eyes, lungs, and cardiovascular system [1]. These extraarticular manifestations occur in up to 40% of patients and, although they may precede polyarthritis, they rarely constitute the first manifestation in individuals without a prior diagnosis [1].

Cardiac involvement is frequent but often clinically silent, affecting as many as 50% of patients [1]. Reported complications include pericarditis, pericardial effusion, myocarditis, coronary vasculitis, diastolic dysfunction, and valvular heart disease. Approximately 40% of patients develop pericardial involvement during the disease course, with about one-third progressing to pericardial effusion, the majority of which remain asymptomatic [1].

Pericarditis is the most common cardiac complication of RA but is seldom the first presentation in previously undiagnosed patients [2]. Only a limited number of such cases have been described in the literature. Although around 15% of RA patients develop symptomatic pericarditis, electrocardiographic or imaging abnormalities may reveal pericardial disease in 20%-50% of cases [3]. Symptomatic disease most often manifests with chest pain or dyspnea and is typically associated with nodular RA or high rheumatoid factor titers [4]. Furthermore, symptomatic pericarditis has been linked to increased mortality in RA [4]. Additional pericardial pathologies include cholesterol pericarditis, effusive-constrictive pericarditis, and cardiac tamponade, which may present in both newly diagnosed and established disease [4]. Consequently, a thorough clinical examination, serological testing, and exclusion of alternative causes such as infection, malignancy, or hypothyroidism are crucial for accurate diagnosis [3]. Prompt recognition and targeted management of pericarditis are key to improving outcomes in RA patients [3].

Among the RA patients with pericardial effusion, up to 13% of patients demonstrate silent pericardial involvement on transoesophageal echocardiography [5]. A cross-sectional study of 80 patients showed that pericardial thickening was present in 12.5% of anti-CCP-positive individuals, whereas none of the anti-CCP-negative patients exhibited such findings [6]. The same study also revealed that mild pericardial effusions occurred four times more frequently in the anti-CCP-positive group [6]. Clinically evident pericardial disease is more commonly associated with RF positivity, while higher titers of anti-CCP antibodies, a highly specific marker, correlate strongly with overall disease activity [5]. In our case, the patient was positive for both RF and anti-CCP antibodies, supporting the association described in the literature.

Restrictive pericarditis and tamponade arising from RA-related pericardial effusions are uncommon but potentially life-threatening complications [7]. Their rarity is reflected in the limited literature, which provides little consensus on prognosis or optimal management strategies [8]. Reported cases describe successful outcomes with nonsteroidal anti-inflammatory agents, corticosteroids, and other immunosuppressive therapies [8]. In rapidly progressive disease leading to large effusions and hemodynamic compromise, corticosteroid therapy alone is insufficient, and urgent pericardiocentesis becomes necessary [9]. The pericardiocentesis should be considered both a life-saving intervention and a means of establishing diagnosis in acute tamponade. In parallel, systemic corticosteroids and immunosuppressive therapy remain essential to control the underlying inflammatory process and prevent recurrence [8].

This case demonstrates the rare but life-threatening occurrence of rapid pericardial effusion and cardiac tamponade in uncontrolled rheumatoid arthritis. It highlights the importance of maintaining a high index of suspicion for cardiac involvement in RA, particularly in patients with elevated anti-CCP titers and RF, even when joint disease appears quiescent. The patient’s clinical course was further complicated by DKA. It was likely that in her case, the DKA might have been triggered by rapidly progressing pericardial effusion with impending cardiac tamponade. There are only a few case reports in the literature where cardiac tamponade was considered as a trigger for DKA [9,10]. Prompt echocardiographic evaluation and urgent pericardial drainage are essential in such emergencies. Additionally, this case underscores the challenges of managing RA alongside comorbidities, emphasizing the need for vigilant monitoring and timely intervention in high-risk patients.

## Conclusions

Rapid pericardial effusion and cardiac tamponade are rare but potentially fatal extra-articular manifestations of uncontrolled rheumatoid arthritis. This case underscores the importance of careful monitoring, timely intervention, and comprehensive management of comorbidities in high-risk RA patients, particularly in patients with elevated anti-CCP titers and RF, even in the absence of active joint symptoms. Early recognition through echocardiography and urgent pericardial drainage can be life-saving in cardiac tamponade. Finally, a high index of suspicion should be maintained, and a thorough search for rare DKA triggers, including cardiac tamponade, should be made when there are no obvious common triggers.
